# Supplementary material for: Bayesian refinement of protein structures and ensembles against SAXS data using molecular dynamics
Source: PLoS Comput Biol. 2017 Oct 18;13(10):e1005800. doi: 10.1371/journal.pcbi.1005800 (PMC5662244; doi:10.1371/journal.pcbi.1005800)
Supplement: S1 Archive — (BZ2) [file pcbi.1005800.s011.tar.bz2 › gromacs_4.62_bayesian_saxs_refi/share/html/online/g_dielectric.html]

g\_dielectric


| g\_dielectric Main Table of Contents |

**VERSION 4.6  
Sat 19 Jan 2013**

---

### Description

g\_dielectric calculates frequency dependent dielectric constants
from the autocorrelation function of the total dipole moment in
your simulation. This ACF can be generated by g\_dipoles.
The functional forms of the available functions are:

One parameter: y = exp(-a\_1 x),  
Two parameters: y = a\_2 exp(-a\_1 x),  
Three parameters: y = a\_2 exp(-a\_1 x) + (1 - a\_2) exp(-a\_3 x).  
Start values for the fit procedure can be given on the command line.
It is also possible to fix parameters at their start value, use -fix
with the number of the parameter you want to fix.

Three output files are generated, the first contains the ACF,
an exponential fit to it with 1, 2 or 3 parameters, and the
numerical derivative of the combination data/fit.
The second file contains the real and imaginary parts of the
frequency-dependent dielectric constant, the last gives a plot
known as the Cole-Cole plot, in which the imaginary
component is plotted as a function of the real component.
For a pure exponential relaxation (Debye relaxation) the latter
plot should be one half of a circle.

### Files

| option | filename | type | description |
| --- | --- | --- | --- |
| **-f** | dipcorr.xvg | Input | xvgr/xmgr file |
| **-d** | deriv.xvg | Output | xvgr/xmgr file |
| **-o** | epsw.xvg | Output | xvgr/xmgr file |
| **-c** | cole.xvg | Output | xvgr/xmgr file |

### Other options

| option | type | default | description |
| --- | --- | --- | --- |
| **-[no]h** | bool | no | Print help info and quit || **-[no]version** | bool | no | Print version info and quit || **-nice** | int | 19 | Set the nicelevel || **-b** | time | 0 | First frame (ps) to read from trajectory || **-e** | time | 0 | Last frame (ps) to read from trajectory || **-dt** | time | 0 | Only use frame when t MOD dt = first time (ps) || **-[no]w** | bool | no | View output .xvg, .xpm, .eps and .pdb files || **-xvg** | enum | xmgrace | xvg plot formatting: xmgrace, xmgr or none || **-[no]fft** | bool | no | use fast fourier transform for correlation function || **-[no]x1** | bool | yes | use first column as x-axis rather than first data set || **-eint** | real | 5 | Time to end the integration of the data and start to use the fit || **-bfit** | real | 5 | Begin time of fit || **-efit** | real | 500 | End time of fit || **-tail** | real | 500 | Length of function including data and tail from fit || **-A** | real | 0.5 | Start value for fit parameter A || **-tau1** | real | 10 | Start value for fit parameter τ1 || **-tau2** | real | 1 | Start value for fit parameter τ2 || **-eps0** | real | 80 | ε0 of your liquid || **-epsRF** | real | 78.5 | ε of the reaction field used in your simulation. A value of 0 means infinity. || **-fix** | int | 0 | Fix parameters at their start values, A (2), tau1 (1), or tau2 (4) || **-ffn** | enum | none | Fit function: none, exp, aexp, exp\_exp, vac, exp5, exp7, exp9 or erffit || **-nsmooth** | int | 3 | Number of points for smoothing |

---

http://www.gromacs.org  
gromacs@gromacs.org
